# Successful management of bile duct injury with duodenal perforation using endoscopic naso-pancreatic drainage and fully covered self-expandable metallic stent deployment

**DOI:** 10.1016/j.vgie.2023.10.011

**Published:** 2023-10-28

**Authors:** Haruka Okada, Eisuke Iwasaki, Seiichiro Fukuhara, Masayasu Horibe, Motoki Sasaki, Takanori Kanai, Naohisa Yahagi, Motohiko Kato

**Affiliations:** 1 Division of Gastroenterology and Hepatology, Department of Internal Medicine, Keio University School of Medicine, Tokyo, Japan; 2 Center for Diagnostic and Therapeutic Endoscopy, Keio University School of Medicine, Tokyo, Japan; 3 Division of Research and Development for Minimally Invasive Treatment, Cancer Center, Keio University School of Medicine, Tokyo, Japan

## Abstract

Video 1 A case of bile and pancreatic duct injury with duodenal perforation during endoscopic submucosal dissection for superficial duodenal epithelial neoplasia.

A case of bile and pancreatic duct injury with duodenal perforation during endoscopic submucosal dissection for superficial duodenal epithelial neoplasia.

## Introduction

Due to improvements in endoscopic techniques, the opportunity to perform endoscopic submucosal dissection (ESD) for periampullary adenoma has gradually increased. However, it remains a challenging procedure even in high-volume centers. The duodenum’s anatomical characteristics, such as the fixed position to the retroperitoneum, tortuous lumen, thin duodenal wall, and rich Brunner’s glands in the submucosa, make ESD difficult. Additionally, exposure to bile or pancreatic fluid may increase the risk of delayed bleeding and perforation. In clinical practice, the incidence of perforation and bleeding is reportedly 13% to 50% and 20%, respectively.^1^

Biliary self-expandable metallic stent (SEMS), pancreatic stent deployment after endoscopic papillectomy,^2^ and endoscopic naso-pancreatic drain placement after ESD involving the papilla have been reported as preventive strategies for postprocedural adverse events.^3^ Here, we report a case in which simultaneous bile duct injury and duodenal perforation were successfully managed by placing a SEMS and naso-pancreatic drain.

## Case

**Figure 1:**
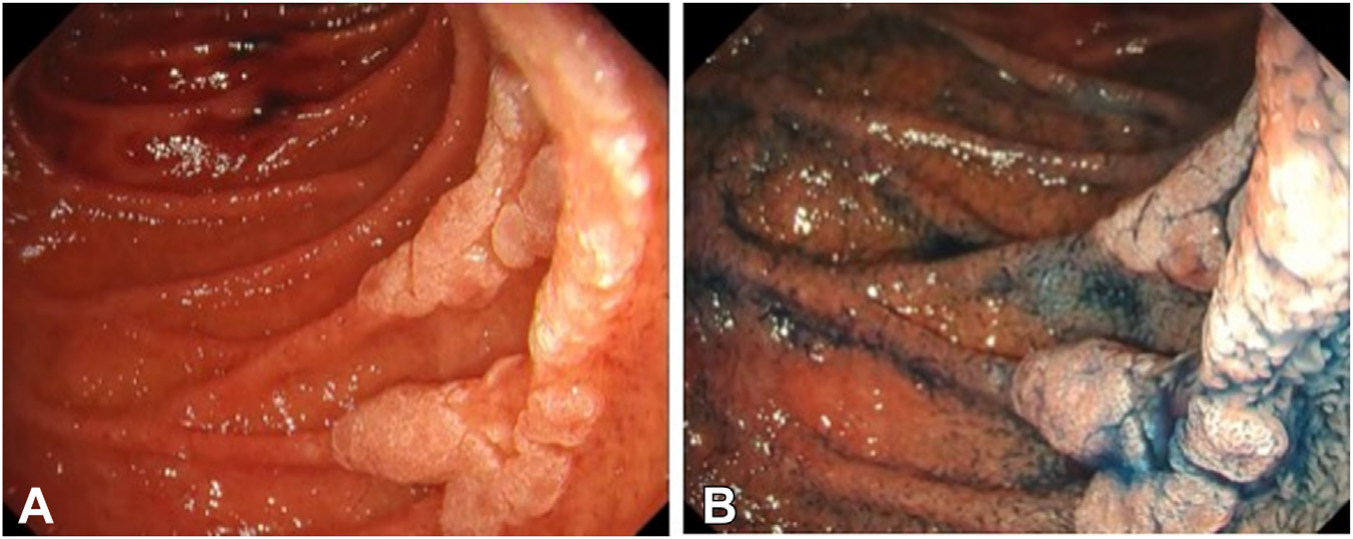
Workup endoscopic images. **A,** Large, flat-elevated tumor located in the second portion at the oral side of the papilla. **B,** Chromoendoscopy with indigo carmine emphasizes elevated nodules on the tumor.

**Figure 2:**
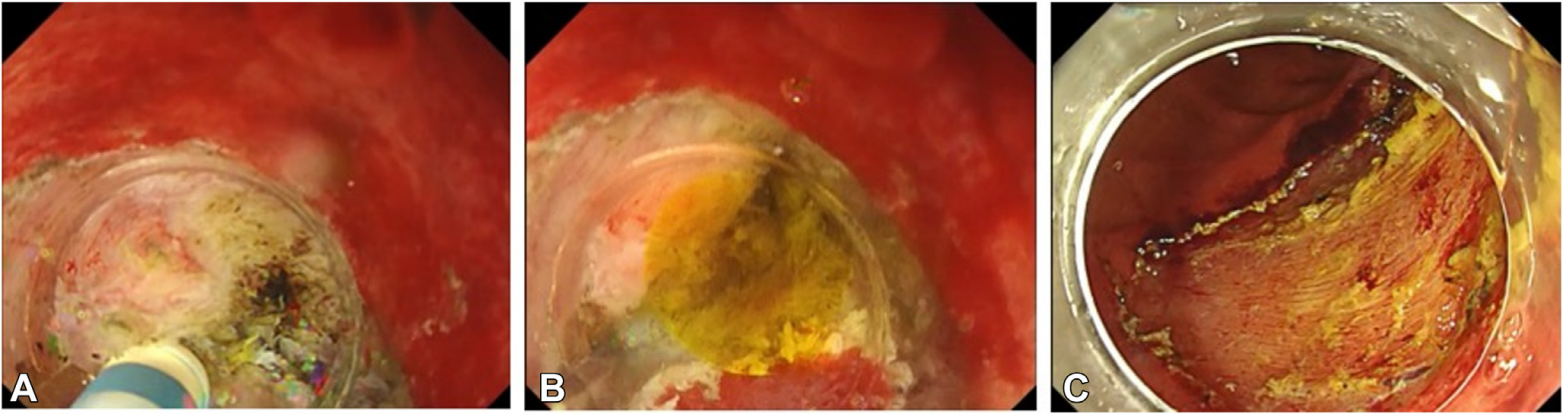
Endoscopic images of endoscopic submucosal dissection. **A,** Endoscopic submucosal dissection is performed using a dual knife with the water pressure method. **B,** Bile duct injury is identified as a result of bile leakage during submucosal dissection. **C,** Tumor is completely dissected.

**Figure 3:**
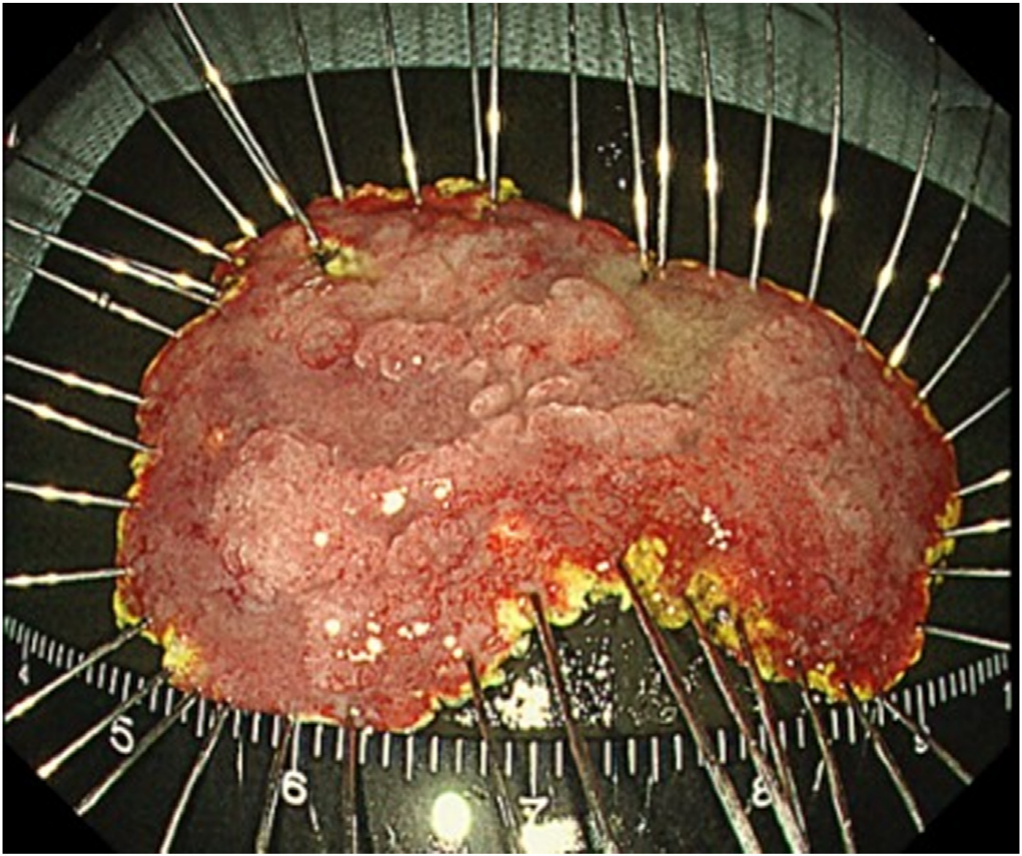
Endoscopic image of the specimen (49 × 37 mm in size).

**Figure 4:**
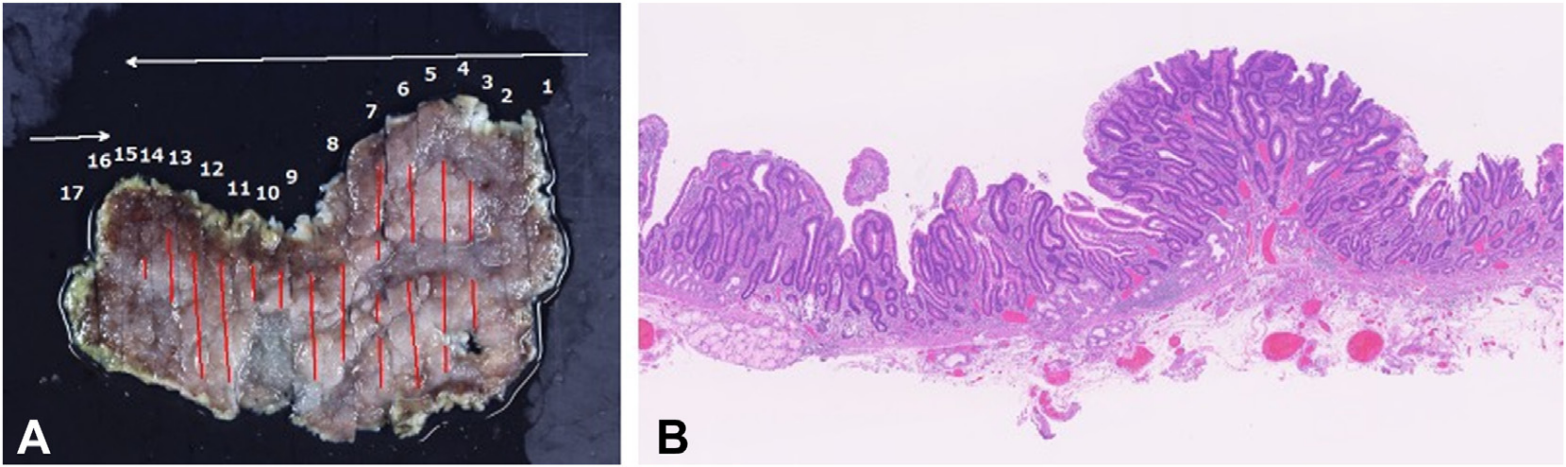
Histopathologic findings. **A,** Histopathologic mapping of the specimen. The adenoma lesion is marked in *red*. **B,** Histopathologic examination of the specimen with a loupe (H&E, orig. mag. ×10). The tumor is diagnosed as tubular adenoma, low grade, intestinal type with complete en bloc resection.

**Figure 5:**
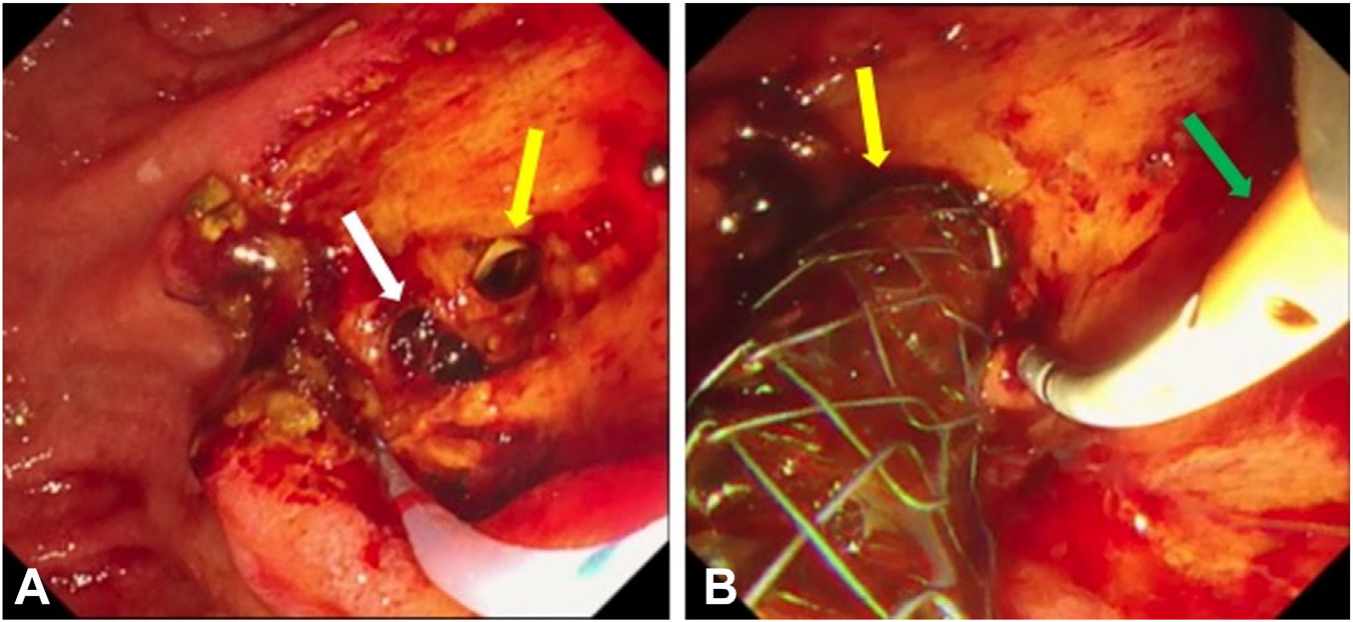
Endoscopic images of ERCP. **A,** The perforation site (*white arrow*) and the bile duct with lateral transection (*yellow arrow*) are identified. **B,** Fully covered self-expandable metallic stent (*yellow arrow*) for bile duct and endoscopic submucosal dissection (*green arrow*) for pancreatic duct are deployed.

An asymptomatic 56-year-old male patient presented with a large periampullary lesion and was referred to our center for endoscopic treatment. Workup endoscopy revealed a 50-mm, flat, elevated lesion located in the second portion on the oral side of the papilla (Fig. 1). The preoperative diagnosis was an adenoma, and we adopted ESD for safety, to secure en bloc resection, and for accurate pathologic diagnosis.^4^ ESD was performed using a DualKnifeJ (Olympus, Tokyo, Japan) with the water pressure method^5^ with the patient under conscious sedation (Fig. 2A). Although this was technically challenging because of the narrow submucosal space to dissect due to rich Brunner’s glands, en bloc resection was achieved in 146 minutes (Fig. 2C). The resected tumor was 49 × 37 mm in size, with pathologic findings of low-grade tubular adenoma (Figs. 3 and 4). During the procedure, extensive bile juice leakage was observed (Fig. 2B); therefore, we carefully observed the wound using a side-viewing endoscopy. Consequently, the accidental bile duct disconnection with a retroperitoneal perforation on the dorsal side near the sphincter of Oddi was confirmed (Fig. 5A).

**Figure 6:**
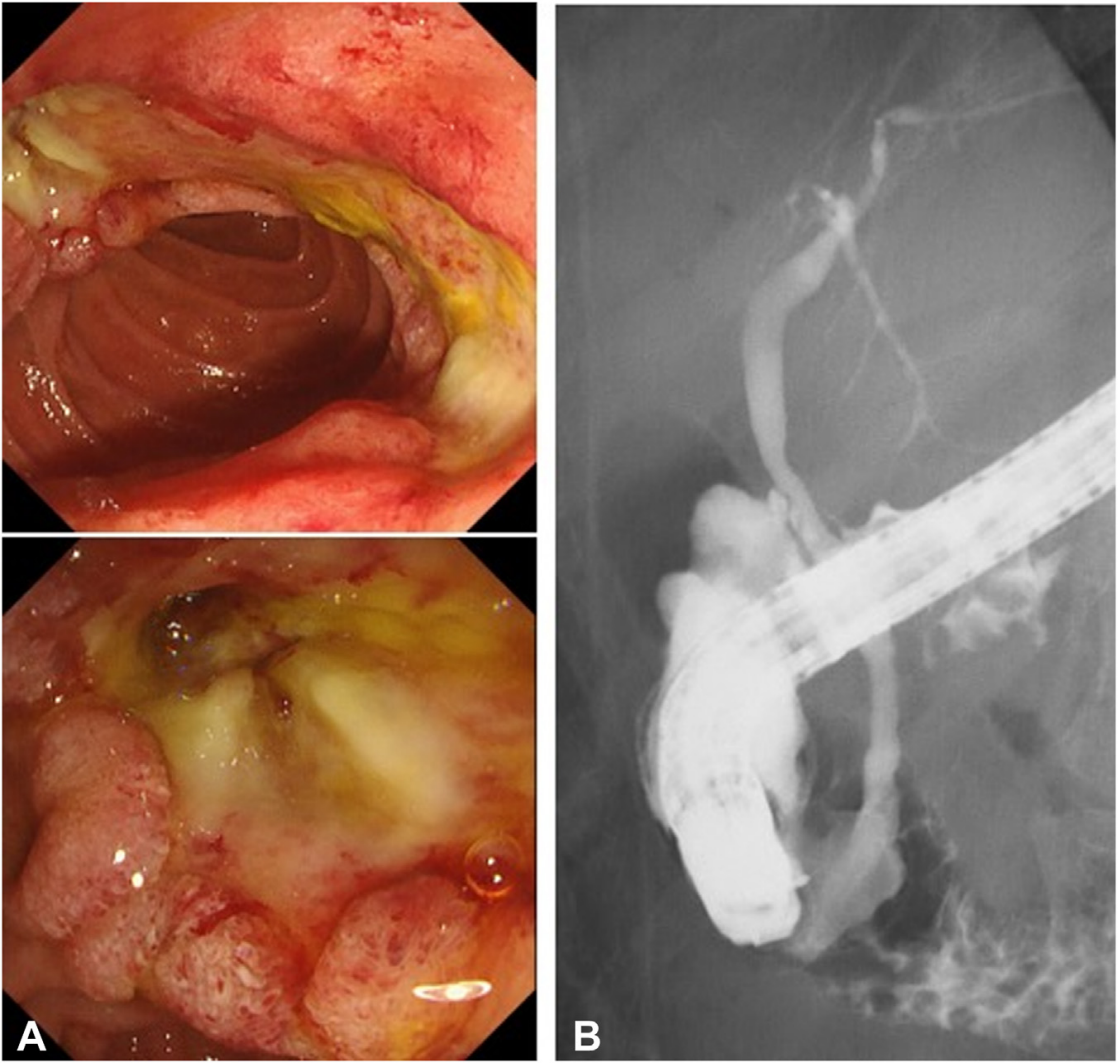
Follow-up images after removal of self-expandable metallic stent and endoscopic submucosal dissection on postoperative day 8. **A,** Endoscopy shows that the post–endoscopic submucosal dissection ulcer has shrunk, and the perforation has completely closed. **B,** Fluoroscopy showing the absence of retroperitoneal leakage of contrast medium.

The transection surface of the bile duct was relatively large to place a naso-biliary drain tube. Subsequently, we deployed a fully covered SEMS for the bile duct in preference to suturing the wound and a naso-pancreatic drain for the pancreatic duct after the insertion of guidewires into the biliary and pancreatic ducts (Fig. 5B).^6^ The patient experienced mild peritonitis with retroperitoneal perforation after the procedure, and the volume of naso-pancreatic drainage was adequate at approximately 200 mL daily without suction. He was treated conservatively with the backup of surgery, the infusion of antibiotics, proton pump inhibitor (20 mg of omeprazole daily), octreotide, and fasting for 8 days. Follow-up esophagastroduodenoscopy on postoperative day 8 showed that the post-ESD ulcer had shrunk, and the perforation had completely closed. After confirming the absence of retroperitoneal leakage of contrast medium, the SEMS and naso-pancreatic drain were removed (Fig. 6). Finally, the patient was discharged without any subsequent adverse events after a 12-day postoperative hospitalization (Video 1, available online at www.videogie.org).

## Conclusion

The endoscopic treatment of periampullary adenoma close to ampullary lesions remains challenging. Although bile duct injury during ESD is a rare adverse event of ESD, extreme caution is required, particularly when resecting lesions located on the oral side of the papilla. Therefore, surgical intervention can be avoided by completely isolating the perforated site and pancreatic juice using a naso-pancreatic drain and compressing the perforated site by deploying a fully covered SEMS.

## Disclosure

Dr Iwasaki received grant support from Gadelius Medical. All other authors disclosed no financial relationships relevant to this publication.

## Supplementary data

Video 1 A case of bile and pancreatic duct injury with duodenal perforation during endoscopic submucosal dissection for superficial duodenal epithelial neoplasia.
